# The neglected epidemic of type 5 diabetes mellitus

**DOI:** 10.1097/MS9.0000000000003985

**Published:** 2025-09-30

**Authors:** Zarmeen Azhar, Zehra Sadia, Araj Naveed Siddiqui, Hermann Yokolo

**Affiliations:** a Karachi Metropolitan University, Karachi, Pakistan; b Jinnah Sindh Medical University, Karachi, Pakistan; c Department of Research, Medical Research Circle (MedReC), Goma, DR Congo

**Keywords:** malnourishment, malnutrition-related diabetes, neglected epidemic, type 5 diabetes

## Abstract

There are several types of diabetes, but type 1 and type 2 often dominate the discussion. They overshadow lesser-known types like type 5 diabetes (T5DM). T5DM was first identified in 1955 in Jamaica. However, it remained understudied due to limited evidence. Recently, in April 2025, the International Diabetes Federation brought attention to this condition during the World Diabetes Congress. It is estimated to affect 20 to 25 million people, predominantly of Asian and African descent. It disproportionately affects malnourished teens in low- and middle-income countries and is therefore, also known as malnutrition-related diabetes. Despite existing for over 70 years, it remains often misunderstood or misdiagnosed. This may lead to inadequate management of increased risk of complications. This highlights the need for reliable diagnostic criteria and extensive research. Furthermore, we believe addressing type 5 diabetes requires a multifaceted, global approach.

Diabetes is a chronic disease that affects 11.1% of the global population, approximately 583 million people, or 1 in every 9 adults aged 20–79 years. This highlights the growing burden of the disease, which is thought to increase by 46% (853 million) by 2050 ^[1]^. While type 1, type 2, and gestational diabetes are the most common, other forms of diabetes account for approximately 1.5–2% of cases^[2]^. Among the most common varieties, type 2 diabetes mellitus (T2DM) accounts for over 90% of cases ^[1]^. These well-known classifications, however, overshadow a less recognized but potentially significant sub-type, type 5 diabetes mellitus (T5DM)^[3,4]^.

On 8 April 2025, during the World Diabetes Congress, the International Diabetes Federation (IDF) introduced a new type of diabetes, namely malnutrition-related diabetes or type 5 diabetes. This new type was initially recognized in 1955 in Jamaica and later officially recognized by the World Health Organization (WHO) in 1985, but was eventually removed due to a lack of evidence^[5]^. This article complies with the TITAN 2025 Guidelines – repressing the reporting and use of artificial intelligence (AI)^[6]^.

T5DM is estimated to affect 20–25 million people worldwide, especially in regions of Asia and Africa. It primarily affects lean and malnourished teens and young adults of low- and middle-income countries. It is thought to arise from chronic malnutrition during early childhood and adolescence, which leads to severe insulin deficiency. For this reason, it is also known as severe insulin-deficient diabetes^[7]^. The pathophysiology of T5DM is multi-factorial and not fully understood, but several mechanisms have been proposed. However, it is widely believed that chronic malnutrition impairs the development and function of pancreatic beta cells, leading to reduced insulin secretion and, ultimately, T5DM^[8]^. This type of diabetes is characterized by pancreatic fibrosis, pancreatic atrophy, and insulin deficiency^[9]^.

It is essential to distinguish T5DM from other types of diabetes. Unlike type 1 diabetes, caused by autoimmune destruction of pancreatic beta cells, and type 2 diabetes, marked by insulin resistance, type 5 diabetes arises from malnutrition during critical stages of development^[7]^. Additionally, malnutrition-induced alterations in the gut microbiome may exacerbate the metabolic dysfunction that is seen in T5DM^[10]^.

Although T5DM has been prevalent for over 70 years, it is often overlooked in global health-related discussions, which has led to its misdiagnosis as type 1 or type 2 diabetes^[1,7]^. Another reason is that the diagnostic criteria for T5DM are not well-established, contributing to its under-diagnosis and misclassification. This may result in inappropriate clinical management and an increased risk of long-term complications, ultimately leading to higher mortality rates.

In conclusion, type 5 diabetes, or malnutrition-related diabetes, is a critical and often overlooked health issue affecting a significant portion of the global population. Further research is needed to establish reliable diagnostic criteria to differentiate T5DM from other types of the disease. Addressing T5DM requires a multifaceted approach, including improving nutritional status, promoting early detection and accurate diagnosis, and developing tailored treatment strategies. Public health interventions aimed at preventing malnutrition, particularly in vulnerable populations, are crucial for reducing the incidence of T5DM^[11]^. Furthermore, adopting therapeutic interventions similar to those used to reduce the risk of progression from prediabetes to type 2 diabetes mellitus^[12]^ could prove beneficial, especially when tailored for different populations.

## Data Availability

Not applicable.

## References

[1] International Diabetes Federation. Facts & figures. 2025. Accessed 8 August 2025. https://idf.org/about-diabetes/diabetes-facts-figures/

[2] International Diabetes Federation. Rare forms of diabetes. 2025. Accessed 8 August 2025. https://idf.org/about-diabetes/rare-forms-of-diabetes/

[3] Hassan K Alghamdi MA Althagafi WE. Application of new modifying models and lifestyle changes and its role in providing better treatment for diabetes mellitus. Int J Commun Med Public Health 2020;7:4146.

[4] Siddique S Zhang G Kubwabo C. Exposure to bisphenol a and risk of developing type 2 diabetes: a mini review. Emerg Contam 2020;6:274.

[5] Medscape Medical News. Malnutrition-Related Diabetes Officially Named ‘Type 5’. 2025. Accessed 10 August 2025. https://www.medscape.com/viewarticle/malnutrition-related-diabetes-officially-named-type-5-2025a10008pd

[6] Agha R Mathew G Rashid R. Transparency in the reporting of artificial intelligence – the TITAN guideline. Prem J Sci 2025;10:100082.

[7] International Diabetes Federation. IDF launches new type 5 diabetes working group. 2025. Accessed 10 August 2025. https://idf.org/news/new-type-5-diabetes-working-group/

[8] Rao RH. The Role of Undernutrition in the Pathogenesis of Diabetes Mellitus. Diabetes Care 1984;7:595. 6439534 10.2337/diacare.7.6.595

[9] Ge S Yang M Cui Y. The clinical characteristics and gene mutations of maturity-onset diabetes of the young type 5 in sixty-one patients. Front Endocrinol (Lausanne) 2022;13. doi:10.3389/fendo.2022.911526 PMC9281895 35846334

[10] Xuan L Keita W, and Ikuo K. Gut microbiota dysbiosis drives and implies novel therapeutic strategies for diabetes mellitus and related metabolic diseases. Front Immunol 2017;8:1882. 29326727 10.3389/fimmu.2017.01882 PMC5742320

[11] Ozougwu O. The pathogenesis and pathophysiology of type 1 and type 2 diabetes mellitus. J Physiol Pathophysiol 2013;4:46.

[12] Hsueh WA McLellan KCP Wyne K. Therapeutic interventions to reduce the risk of progression from prediabetes to type 2 diabetes mellitus. Ther Clin Risk Manag 2014;10:173–188. 24672242 10.2147/TCRM.S39564 PMC3964168

